# Rapid antigen testing for COVID-19: Decreasing diagnostic reliability, potential detrimental effects and a lack of evidence to support continued public funding of community-based testing

**DOI:** 10.1016/j.puhip.2023.100451

**Published:** 2023-11-23

**Authors:** Tracy Beth Høeg, Vinay Prasad

**Affiliations:** a Department of Epidemiology and Biostatistics, University of California-San Francisco, USA; b Department of Clinical Research, University of Southern Denmark, Odense, Denmark

## Abstract

Rapid antigen testing continues to be broadly recommended across the world for the prevention of transmission of SARS-CoV-2. We explore existing recommendations in the United States, evidence of decreasing diagnostic reliability of individual tests and potential benefits and harms of non-targeted testing. Recent research has found multiple commonly-used rapid antigen tests to now have diagnostic sensitivities below 30%, with sensitivities at or near 0% the first 48 hours of infection, using polymerase chain reaction (PCR) test positivity as the gold standard. Reliance on tests with low sensitivity could paradoxically increase transmission risk through false assurance. Furthermore, widespread testing has substantial direct and indirect costs, while its effectiveness for diminishing COVID-19 disease burden or improving overall community health is unclear. Because benefit has not been demonstrated with high-quality evidence, we argue against 1. The continued recommendations for and 2. Public funding of widespread community-based rapid antigen testing.

## 1 Introduction

Rapid antigen testing for COVID-19 continues to be broadly recommended by the Centers for Disease Control and Prevention (CDC) in United States for anyone with COVID-19 symptoms, recent exposure [1], those visiting high-risk individuals or those recently in crowded spaces while traveling without wearing a mask [2]. In September of 2023, the Biden administration dedicated an additional $600 million to provide four rapid antigen tests free of charge to all US households [3]. The National Institutes of Health (NIH)'s current information [4] on COVID-19 testing notes, “testing is critical to controlling the spread of SARS-CoV-2” and is “the only way to be sure you are not passing the virus on to others.” The NIH has provided specific grant money [4] to increase access to rapid antigen testing in underserved communities and states “frequent testing may help end the pandemic.” Also in 2023, the Infectious Disease Society of America (IDSA) recommended [5] “broader testing strategies” to the Biden administration. Meanwhile, already in early 2022, Scandinavian countries abandoned recommending population-based testing except in situations where a positive result can change medical management [5,6]. In this review, we critically appraise the current utility of community-based rapid antigen testing for COVID-19.

## 2 COVID-19 testing: A history of costs, benefits and unintended consequences

Just one rapid antigen testing company, Abbott, was estimated to have made over $15 billion in the US in 2021 and 2022 combined [7]. As of January 2022, the US government had committed over $4 billion for the development and production of rapid antigen testing [8]. In 2022, the US goverment spent an additional $2 billion on free rapid antigen tests for all households in the US [20] and then dedicated another $600 million to the same in 2023[3]. By the start of 2023, Medicare had spent over $5.5 billion to cover rapid antigen testing. [21]As of November 2023, over-the-counter rapid tests continue to be covered by Medicaid and may still be covered by Medicare Advantage. [22] Private insurance companies are also still required to reimburse up to $12 for each rapid antigen test, [23] which may result in increased insurance premiums and/or increasing co-pays.

With the emergence of the omicron variant and increasing population immunity, the infection hospitalization and infection fatality rate of COVID-19 have diminished substantially [9]. Along with this, the theoretical benefit of any population-based testing program has also diminished. Determining the cost-benefit ratio of such a program is also incredibly complex [10] and real-life analyses demonstrate the importance of considering unintended consequences, such as increased school and work absences, unnecessary medicalization and inaccurate test results [11]. The potential benefits of population-based testing programs will also depend on the likelihood a person will quarantine if the test is positive and how many deaths, hospitalizations and lost work days due to COVID-19 will be prevented or delayed (and for how long). An individual with a positive test result may not be able to quarantine or only partially quarantine. Some quarantines may not prevent any transmission but come with a loss of productivity and absences from important events. A false negative result may cause a person to expose more individuals to SARS-CoV-2 while a true negative result may lead individuals symptomatic with a different disease to be falsely assured and expose more individuals to a non-COVID illnesses, which could be more severe.

In 2020, testing played an important role in establishing accurate infection fatality rates and provided insight about the transmission of this novel disease. Testing has also been used to. provide individuals information about their own immunity and for alerting high-risk populations about periods of increased community transmission. One modelling study from Italy in 2020 found a population-based rapid testing program may have been cost-effective through slowing transmission rates [12], however this was based on observational data where causality between the testing and decreased disease spread, hospitalizations and deaths could not with certainty be attributed to the testing program as many variables were changing simultaneously. It was also not clear to what extent COVID-19 hospitalizations and deaths, if truly diminished due to testing, may have been entirely prevented and not just delayed.

Particularly now that COVID-19 has become endemic, it is important to consider the limitations and unintended consequences of ongoing testing, especially on non-high-risk groups.

## 3 Diagnostic accuracy of SARS-CoV-2 antigen tests

Rapid antigen tests have had decreasing accuracy in the setting of the omicron variants. There are several factors which influence the diagnostic accuracy of the SARS-CoV-2 rapid antigen tests. These include the sensitivity and specificity of the individual tests, the pretest probability a person is infected with SARS-CoV-2, the circulating variant, the individual's immunity status and the stage of infection.

A Cochrane review [13] of rapid antigen tests published in 2022, based on the pre-omicron era, found an average sensitivity of 54.7 % for people testing without symptoms, 49.6 % for people who are not known contacts. The specificity was on average 99.5 % for all rapid tests. Specifically, Abbot BinaxNOW, the only rapid antigen test in this study currently authorised for use in the US, had a slightly higher sensitivity at 58.7 % and specificity of 99.8 % for asymptomatic participants and 80.9 % sensitivity and 99.9 % specificity for symptomatic patients. Those without symptoms using Abbot BinaxNOW, at a disease prevalence of 0.5 %, had a positive predictive value of only 59.6 % if they had no known contacts. In this situation, over 40 % of the time a positive rapid test will not be truly positive. If an individual was symptomatic and had a 50 % pretest probability, over 16 % of the time a person had a negative result, they would actually be infected.

Studies following the emergence of the omicron variant have found even lower sensitivities of rapid tests, especially early in the infection. One study [14] performed during omicron outbreaks in New York City, Los Angeles and San Francisco using two popular rapid antigen tests (Abbott BinaxNOW and Quidel QuickVue) found 0 % sensitivity within the first 48 h a person is PCR positive. Importantly, this study exclusively considered cases that were believed to be infectious based on a cycle threshold (Ct) of <29. Between 48 and 72 hour post PCR positivity, the sensitivity improved to only 29 %, again using the same Ct cutoff. Specificity could not be calculated as only PCR positive cases were included. Importantly, 14 % of cases with infectious viral load (Ct < 29) were thought to have been linked to onward transmission, all of which occurred before the rapid antigen test turned positive [personal communication of lead author Blythe Adamson with TBH]. This study suggests sensitivity may be lowest, and approaching 0 % with a false negative rate of nearly 100 % during an outbreak and at the early stages of infection, including periods when at least some people are already infectious

A second omicron-specific study [15] examined the BinaxNOW among Stanford University athletes and found an overall sensitivity of only 39.2 % among those who were asymptomatic and 77.8 % among those with symptoms. The specificity was overall high at 99.8 %. For those without symptoms at a disease prevalence of 0.5 %, positive predictive value would be only 49.6 %. However, in a case where a person is symptomatic and there is a pretest probability of 50 %, or very high, about 18 % of the time a person gets a negative result, it will be a false negative. This study did not specifically look at false negative rate over time nor did they explore onward transmission. They did report wide Ct ranges in symptomatic false negatives between 10 and 45, indicating many of these false negatives may have indeed been infectious.

Another study [16] from the Netherlands found an even lower sensitivity of 27.5 % among asymptomatic individuals using the Flowflex rapid antigen test by Acon Laboratories. The sensitivities of multiple test brands were found to be consistently lower among previously-infected individuals. This may explain some of the decreasing sensitivity of rapid antigen testing we are seeing over time. Notably, overall sensitivity increased to 48.3 % when a viral load cutoff was used, above which at least 95 % had a positive viral culture.

A final, more recent study of ten commonly-used rapid antigen tests in the setting the BA.4 and BA.5 omicron subvariants [17] reported even lower sensitivities. Among individuals where symptom status was unknown, using intermediate Ct values of 25-30, sensitivites dropped to 0-26 %. This included tests that had previously had sensitivies nearing 90 % prior to the emergence of the omicron variant.

## 4 Weighing current potential benefits and harms

Individuals who are temporarily immune-compromised will likely benefit during a specified period of time from avoiding exposure to SARS-CoV-2. However, screening of contacts with rapid tests may be misleading, particularly early in the infection. With sensitivities currently under 30% in the setting of the omicron subvariants, and as low as 0% during the initial days of infection, the use of rapid antigen tests is likely to provide a false sense of security for events including high-risk individuals. Symptomatic individuals who test negative may also expose high-risk individuals to non-COVID-19 illness at higher rates. Taking on riskier behavior due to an increased sense of security is referred to as the Peltzman effect or, more simply, risk compensation.

Testing of high-risk individuals can help provide appropriate, disease-specific treatment. Testing of non-high-risk individuals that will not alter treatment could theoretically result in behavior changes, which decrease community transmission rates. However, this has yet to be demonstrated with high-quality data that allow for causal inference. In fact, one retrospective observational study [18] found moderate COVID-19 screening testing rates among college students were associated with the highest student case rates. Though this study was observational in nature, it serves as a reminder that public health programs that have theoretical benefits may not be effective in real life. It also points to the need for higher quality data to justify the recommendation for and use of public funds for community-based non-targeted testing.

Notably, no amount of testing has thus far succeeded in preventing spread of variants globally. Schools or campuses with higher COVID-19 testing rates have not been found to have lower case or hospitalization rates either for COVID-19 or overall. The benefit of delaying infection for a matter of months is also unclear. Behaviour changes related to repeat quarantines may also increase the risks of other health problems. The costs of testing, missed work and school days for quarantines and the emotional toll of missing major life events, particularly for asymptomatic positive test results, should all be considered in any risk-benefit analysis. Finally, in terms of community health, money spent on testing of low risk individuals may be better used for a more comprehensive sick leave program.

## 5 A current lack of evidence of net benefit

Ultimately, determining whether encouraging rapid testing can improve overall community health will require either randomized trials or high-quality observational data which permit causal inference. Communities or counties could be randomized to instructions to test before gatherings and/or free tests. The primary outcome could be SARS-CoV-2 transmission in those areas at 15 or 30 days later or longer-term COVID-19 and all-cause hospitalization rates comparing communities that had and hadn't received tests. Importantly, testing is only useful in so far as its results can leverage behavioral changes that improve outcomes [19]. Based on the aforementioned considerations, a rigorous study may reveal no or even increased risk of transmission or all-cause hospitalization after implementing or recommending a rapid testing program.

## 6 Conclusion

Especially in light of the current data demonstrating decreasing accuracy of rapid antigen testing coupled with decreasing COVID-19 disease severity, we argue better evidence of benefit should be required before the continued broad recommendation and expenditure of public funds on community-based rapid testing. While it is likely that the SARS-CoV-2 rapid antigen tests still maintain some utility in high-risk situations, widespread use of these tests comes with large costs, high rates of false negatives and a documented inability to stop the global spread of new variants. Indiscriminate testing may even paradoxically increase the chance of disease and divert resources from other uses that can have a greater positive impact on community health.

## Funding

None.

## Declaration of competing interest

The authors declare the following financial interests/personal relationships which may be considered as potential competing interests: Vinay Prasad's Disclosures. (Research funding) Arnold Ventures (Royalties) Johns Hopkins Press, Medscape, and MedPage (Honoraria) Grand Rounds/lectures from universities, medical centers, non-profits, and professional societies. (Consulting) UnitedHealthcare and OptumRX. (Other) Plenary Session podcast has Patreon backers, YouTube, and Substack. Tracy Beth Høeg's Disclosures. (Honoraria) Brownstone Institute, Global Liberty Institute. (Consulting) Florida Department of Health (Other) Substack, payment for writing at The Atlantic, Los Angeles Times, New York Times, The Hill, Daily News and Tablet Magazine.

## References

[1] Centers for Disease Control and Prevention. COVID-19 Testing: What you need to know. Updated September 23^rd^, 2023. https://www.cdc.gov/coronavirus/2019-ncov/symptoms-testing/testing.html. Accessed November 7, 2023..

[2] Centers for Disease Control and Prevention. COVID-19. Updated August 22^nd^, 2023. .https://wwwnc.cdc.gov/travel/diseases/covid19.

[3] Department of Health and Human Services (2023). Biden-harris administration awards $600 million to bolster US manufacturing of COVID-19 tests and announces the Re-opening of COVIDTests.gov. https://www.hhs.gov/about/news/2023/09/20/biden-harris-administration-awards-600-million-bolster-us-manufacturing-covid-19-tests-announces-re-opening-covidtestsgov.html.

[4] National Institutes of Health (NIH). COVID-19 Testing. https://covid19.nih.gov/covid-19-topics/covid-19-testing. Accessed November 7th, 2022..

[5] Helse Norge. Coronavirus.https://www.helsenorge.no/en/coronavirus/. Accessed January 9th, 2023..

[6] Danish Health Authority COVID-19 test, symptoms and early treatment. https://sst.dk/en/english/corona-eng/guidelines-vaccination-and-disease-prevention/test-symptoms-and-early-treatment.

[7] Biotech Fierce (July 20, 2022). Abbott Holds onto billion dollar covid test sales a little longer pushing decline predictions to this fall. https://www.fiercebiotech.com/medtech/abbott-holds-billion-dollar-covid-test-sales-little-longer-pushing-decline-predictions-fall#:~:text=In%20April's%20first%2Dquarter%20earnings,three%20months%20of%20the%20year.

[8] Brown KV, Murphy P and Bloomberg Fortune. COVID Test Makers set to make $4 billion in U.S. government work. https://fortune.com/2022/01/21/test-makers-make-4-billion-us-government-work-free-covid-kits/. January 21, 2022..

[9] Erikstrup C., Laksafoss A.D., Gladov J. (2022 Oct). Seroprevalence and infection fatality rate of the SARS-CoV-2 Omicron variant in Denmark: a nationwide serosurveillance study. Lancet Reg Health Eur.

[10] Vilches T.N., Rafferty E., Wells C.R. (2022). Economic evaluation of COVID-19 rapid antigen screening programs in the workplace. BMC Med..

[11] Leng T., Hill E.M., Thompson R.N., Tildesley M.J., Keeling M.J., Dyson L. (2022 May 27). Assessing the impact of lateral flow testing strategies on within-school SARS-CoV-2 transmission and absences: a modelling study. PLoS Comput. Biol..

[12] Cavazza M., Sartirana M., Wang Y., Falk M. (2023 Oct 10). Assessment of a SARS-CoV-2 population-wide rapid antigen testing in Italy: a modeling and economic analysis study. Eur. J. Publ. Health.

[13] Dinnes J., Sharma P., Berhane S. (2022 July 22). Rapid, point-of-care antigen tests for diagnosis of SARS-CoV-2 infection. Cochrane Database Syst. Rev..

[14] Adamson B, Sikka R, Wyllie AL, Premsrirut P. Discordant SARS-CoV-2 PCR and rapid antigen test results when infectious: a December 2021 occupational case series. medRxiv. 10.1101/2022.01.04.22268770. January 5, 2022. [including personal communication about results].

[15] Tsao J., Kussman A.L., Costales C., Pinsky B.A., Abrams G.D., Hwang C.E. (2022). Accuracy of rapid antigen vs reverse transcriptase–polymerase chain reaction testing for SARS-CoV-2 infection in college athletes during prevalence of the omicron variant. JAMA Netw. Open.

[16] Venekamp RP, Schuit E, Hooft L (2023 Mar). Diagnostic accuracy of SARS-CoV-2 rapid antigen self-tests in asymptomatic individuals in the omicron period: a cross-sectional study. Clin Microbiol Infect.

[17] Krenn F., Dächert C., Badell I. (2023). Ten rapid antigen tests for SARS-CoV-2 widely differ in their ability to detect Omicron-BA.4 and -BA.5. Med. Microbiol. Immunol..

[18] Schultes O., Clarke V., Paltiel A.D., Cartter M., Sosa L., Crawford F.W. (2021). COVID-19 testing and case rates and social contact among residential college students in Connecticut during the 2020-2021 academic year. JAMA Netw. Open.

[19] Ioannidis J.P., Prasad V. (2013 Jul 22). Evaluating health system processes with randomized controlled trials. JAMA Intern. Med..

[20] Spolar C. What are taxpayers spending for the 'free' Covid tests? The Government won't say. February 11th, 2022. KFF HealthNews. kffhealthnews.org/news/article/what-are-taxpayers-spending-for-those-free-covid-tests-the-government-wont-say/. Accessed November 24th, 2023.

[21] Damon, A. Medicare keeps spending more on COVID-19 testing. Fraud and overspending are partly why. ProPublica. December 30th, 2022. propublica.org/article/how-fraud-increases-medicare-spending-covid-19-testing. Accessed November 24th, 2023.

[22] Centers for Medicare and Medicaid Services. Coverage for COVID-19 Tests. Testing reamins an important tool in combatting the spread of COVID-19. April, 2023. www.cms.gov/files/document/covid-over-counter-test-coverage.pdf. Accessed November 24th, 2023.

[23] Centers for Medicare and Medicaid Services. How to get your At-Home Over-The-Counter COVID-19 Test for Free. September 6th, 2023. www.cms.gov/how-to-get-your-at-home-OTC-COVID-19-test-for-free. Accessed November 24th, 2023.

